# Modelling the Interaction between Carboxylic Acids and Zinc Oxide: Insight into Degradation of ZnO Pigments

**DOI:** 10.3390/molecules27113362

**Published:** 2022-05-24

**Authors:** Jihan Lubani, Filippo De Angelis, Daniele Meggiolaro, Laura Cartechini, Simona Fantacci

**Affiliations:** 1 Department of Chemistry, Biology and Biotechnology, University of Perugia, UdR INSTM di Perugia, Via Elce Di Sotto 8, 06123 Perugia, Italy; jihanloubani@hotmail.com; 2 Computational Laboratory for Hybrid/Organic Photovoltaics (CLHYO), Istituto CNR Di Scienze e Tecnologie Chimiche “Giulio Natta” (CNR-SCITEC), Via Elce Di Sotto 8, 06123 Perugia, Italy; daniele.meggiolaro@cnr.it (D.M.); laura.cartechini@cnr.it (L.C.); 3 Department of Natural Sciences & Mathematics, College of Sciences & Human Studies, Prince Mohammad Bin Fahd University, Al Khobar 31952, Saudi Arabia

**Keywords:** ZnO pigment, oil paint, degradation, DFT modelling

## Abstract

Computational modelling applied to cultural heritage can assist the characterization of painting materials and help to understand their intrinsic and external degradation processes. The degradation of the widely employed zinc oxide (ZnO)—a white pigment mostly used in oil paints—leads to the formation of metal soaps, complexes of Zn ions and long-chain fatty acids coming from the degradation of the oil binder. Being a serious problem affecting the appearance and the structural integrity of many oil paintings, it is relevant to characterize the structure of these complexes and to understand the reaction pathways associated with this degradation process. Density functional theory (DFT) calculations were performed to investigate the adsorption of the acetate and acetic acid on relatively large ZnO clusters and the formation of Zn–acetate complexes. Carboxylic acids with longer alkyl chains were then investigated as more realistic models of the fatty acids present in the oil medium. In addition, DFT calculations using a periodic ZnO slab were performed in order to compare the obtained results at different levels of theory. Optimization calculations as well as the formation energies of the ZnO@carboxylate coupled systems and the thermodynamics leading to possible degradation products were computed. Our results highlight the potential for DFT calculations to provide a better understanding of oil paint degradation, with the aim of contributing to the development of strengthening and conservation strategies of paintings.

## 1. Introduction

Inorganic pigments have been used since antiquity due to their bright colors and high covering power. Together with oil binder and a variety of additives, inorganic pigments constitute the main components of oil paints. The stability of oil paints is influenced by several factors such as temperature, humidity [1,2,3], and exposure to light [4], pollutants, and solvents [5,6,7]. The oil binding medium, upon exposure to oxygen, first dries via radical polymerization reactions forming a cross-linked molecular network, and then it ages through reactions of the autoxidation products with the pigment and eventually leads to paint degradation [8,9,10,11,12,13,14]. This is a serious problem for paint conservation since degradation can irreversibly change the stability and the appearance of the paint. As an example, zinc oxide (ZnO) is a typical white pigment that degrades in oil by forming metal soaps; these are in general zinc ion complexes with long-chain fatty acids which come from the aging process of the oil binding medium [8]. The consequence of the formation and aggregation of metal soaps is the appearance of small whitish lumps which grow in the underlying paint layer and break through the top surface, resulting in a textured surface marked with jarring spots and transparency of paint layers [8]. The entire ZnO pigment degradation process is a complex reaction pathway that has not yet been completely unravelled since its evolution, as it is very difficult to follow and might also be influenced by external factors [15]. Recent extensive experimental studies on this topic have concluded that the degradation passes through the formation of ionomers and that the final degradation products are zinc soaps, i.e., zinc–carboxylate complexes [16,17,18,19]. It has been hypothesized that ZnO degradation is initialized by the interaction of ZnO pigment with the carboxylic functionalities formed by the autoxidation reactions associated with oil polymerization [20], or with stearic acid, if in the paint tube formulation the jellifying agent AlSt(OH)_2_ has been added, as experimentally observed in a study on degradation in *Alchemy* by J. Pollock [12]. Zinc ions then diffuse from the pigment surface and migrate through the binding medium by ‘hopping’ from one carboxylate group to the neighboring one. These metal ions continue to bind many of the free carboxylic/carboxylate functionalities in the binding medium in a spontaneous manner, thus giving rise to an ionomeric polymeric network [16]. This classification contributes to our understanding of the metal soap formation process. As metal ions are distributed through the binding medium in an ionomer, metal soaps could form where there are free saturated fatty acids available to form a complex [21]. Considering the uncertainty that still exists in the atomistic degradation mechanism of ZnO and its importance for artwork conservation, it is important to clarify the different steps of the ZnO degradation process to help the conservators work.

In this work, we employ advanced computational modeling strategies, based on first-principle electronic structure calculations, to investigate some of potential key steps of the reactive degradation process. Based on the available experimental data on possible degradation pathways, a sequence of plausible intermediates and reaction pathways was calculated to estimate the reaction thermodynamics and to highlight possible mechanisms related to the stability of different degradation products. We first focused on the interaction between ZnO and different oil carboxylic acids that are common degradation products and can adsorb on the ZnO surface. We associated this adsorption step with the initialization of the degradation pathway and then we characterized possible intermediates and final products. We benchmarked different models and methods to calculate the energy associated with selected reactive pathway steps, finding the adsorption of carboxylic acids to ZnO surface and the formation of Zn carboxylates to be thermodynamically favored. Finally, a role of surface acid–base chemistry in determining the energetics of ZnO degradation was proposed, which may be helpful in assisting with pigment protection.

## 2. Models and Computational Methodologies

**Figure 1:**
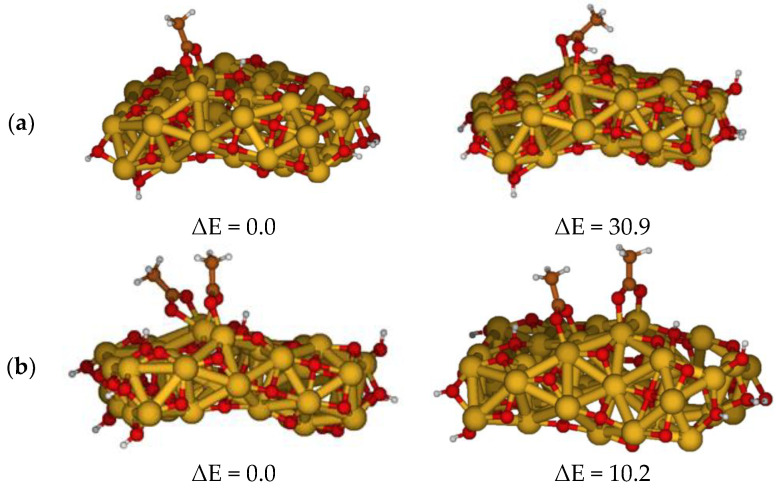
Optimized geometries of possible adsorption configurations in low-polarity medium, considering one acetic acid (**a**) or two molecules of acetic acid (**b**) on the surface, along with the relative energy difference between the configurations in kcal/mol.

Starting from ZnO pigment in its wurtzite crystal structure, we built a cluster which has been already demonstrated as a good model for describing the electronic and optical properties of ZnO and the adsorption of dyes through the carboxylate functionality [22]. The aim of the present paper is to investigate (i) the interaction between ZnO and carboxylic acids and acetates, which are plausible reactive fragments of linseed oil degradation, and (ii) the formation of the ZnO degradation products, the Zn carboxylates. Based on the size of the investigated adsorbates and as a trade off between accuracy and computational efforts, we modelled these reactive processes by employing the acetic acid and a (ZnO)_42_ cluster [22]. The (ZnO)_42_ cluster model shows the wurtzite structure, and the Zn- and O-terminated surfaces were saturated with 4 dissociated water molecules, which was previously shown to be an adequate model to study carboxylic anchoring dye groups adsorption [22]. We considered both protonated acetic acid and acetate anions to adsorb to ZnO on the apolar (1010) surface, see Figure 1, which are the main surfaces exposed to ZnO, even though they are less reactive than the polar ones. We chose acetic acid and acetate since they are the smallest carboxylic acid/carboxylate models representative of longer-chain acids. To evaluate how the acid chain length affects the acid–ZnO interaction and complexation, we also considered hexanoic acid to carry out a comparative analysis. The calculations were performed by the Gaussian 09 program package [23]. The geometry of all the species involved in the considered reactive processes was optimized by means of Density Functional Theory, using the B3LYP exchange–correlation functional [24,25], a DGDZVP basis set [26,27] both in vacuo and in water solution. Solvation effects were included by means of the conductor-like polarizable continuum model (CPCM), as implemented in Gaussian09 [28]. Furthermore, to simulate a low-polarity environment embedding the model cluster, a CPCM model with a dielectric constant of 5 was considered, a value included in the range of dielectric constant values measured for the linseed oil [29]. The energy associated with the investigated reactions was computed in vacuum phase, in water and in the low-polarity medium described above. To benchmark our cluster calculations, we carried out periodic ZnO slab simulations on two acetic acid adsorption modes using the CP2K-6.1 program package within periodic boundary conditions. The PBE exchange–correlation functional was used in these calculations for the sake of efficiency [30,31].

## 3. Results and Discussion

### 3.1. Interaction between Acetic Acid/Acetate and ZnO

To gain insight into the ZnO degradation, we started by investigating the adsorption of the acetic acid (AcH)/acetate (Ac) to the ZnO surface. The coordination of acetic acid/acetate to undercoordinated surface Zn atoms through the carboxylic oxygen atoms can be considered as the initialization of the reaction pathway that leads to the metal soap formation [32]. The optimized structures for single and double AcH adsorption are reported in Figure 1. 

For single AcH adsorption, we explored different adsorption modes (monodentate, bidentate with the proton on the acid or transferred to the surface, and dissociative bidentate) to study their corresponding stability. We focused on the geometries optimized in the low-polarity medium, discussing the geometries optimized in vacuo and in water solvent when marked differences were presented. Water solvents are a high-polarity medium and stable results were found for the adsorption geometries optimized in it, in agreement with previous studies [33,34], and are interesting since humidity is one of the factors that may trigger pigment reactivity in paintings. The most stable optimized configuration shows the acetic acid adsorbed on two adjacent Zn centers by a bridged bidentate configuration transferring the proton to an oxygen of the ZnO surface, therefore in a dissociative adsorption mode, consistent with previous experimental and theoretical studies using different methodological approaches [35,36,37,38,39,40,41,42,43,44]. The monodentate and bidentate AcH adsorption modes involving the same Zn center were found to relax to the dissociative bridged bidentate configuration. The non-dissociative bidentate adsorption was 30.9 kcal/mol higher in energy than the global dissociative minimum, Figure 1a, indicative of the basic nature of ZnO, which is prone to receiving protons from carboxylic acids. The Zn–O bond distances have been optimized (ca. 2.00 and 2.02 Å) for the dissociative configuration, and longer (ca. 2.34 and 2.38 Å) for the non-dissociative one. We also optimized another less stable bridged bidentate adsorption configuration which differs in the position of the two Zn centers involved in the adsorption (see Supplementary Material Figure S1). 

We further considered the adsorption of two AcH units on the ZnO surface; Figure 1b shows two optimized geometries of (AcH)_2_@ZnO dissociative bridged bidentate modes differing in the position of the Zn centers involved in the adsorption of carboxylic acids. In the most stable configuration (Figure 1b, to the left), the central Zn coordinates oxygen atoms of both acids. The Zn–O bond distances are 2.24 and 2.17 Å when the two oxygen atoms are bound to the same Zn, and 2.09 and 2.04 Å when the oxygen atoms are coordinated to different Zn atoms. The higher stability of the adsorption mode suggests that the adsorption of two carboxylic acids on the same Zn ion is favored, a fact which could have implications for the subsequent reactivity of adsorbed species, as discussed below.

We repeated the geometry optimizations for the acetate species Ac^−^, considering the following adsorption systems: [Ac@ZnO]^−^ and [Ac_2_@ZnO]^2−^ in both configurations. [Ac@ZnO]^−^ shows the same bidentate configuration found for the acid, see Figure S2, while for [Ac_2_@ZnO]^2−^ system, in the geometry optimized in solution, the Zn–O bond was detached from the surface; see Supporting Information, Figure S2. The computed Zn–O bond distances are 2.02, 2.8, and 2.09 Å for this configuration and 2.08, 2.09, and 2.10 Å for the other configuration. We therefore observed that for the charged [Ac_2_@ZnO]^2−^ embedded in both solvents of different polarities, the most stable configuration was that with the two coordinated acetate united on different Zn couples. 

We focused on the energetics associated with the binding of one and two molecules of acetic acid/acetate to the ZnO surface, which gives indications on the occurrence of these processes. They lead to the most stable AcH@ZnO and [Ac@ZnO]^−^ (reactions 1 and 2), and (AcH)_2_@ZnO and [Ac_2_@ZnO]^2−^ (reactions 3 and 4).

The reactions 1–4 were exothermic, proving that the acetic acid and acetate, depending on the environmental conditions, chemically adsorb on the ZnO surface, and that these adsorption systems might be plausible intermediates of the global reaction pathways of the degradation. We observed that by increasing the polarity of the medium, the adsorption process is less exothermic (see SI, Table S1); the values in vacuo are overestimated, not describing realistic conditions. Moreover, by comparing the ΔE of the reactions, we found that the adsorption systems associated with acetic acid are more favored than those adsorbing to acetate both in a low-polarity medium and in water. They also present different adsorption geometries. The computed adsorption energy of acetic acid on ZnO surface accounts for both the proton dissociation from the acid and the adsorption of acetate and proton on the surface. The higher exothermicity of the adsorption involving acetic acid with respect to acetate is therefore the result between the energy of the proton dissociation (weakly endothermic step) and that of the proton adsorption (highly exothermic step). Moreover, the adsorption of the proton on the ZnO surface might play a role in favoring metal migration by stabilizing the generated metal vacancy. 

**Figure 2:**
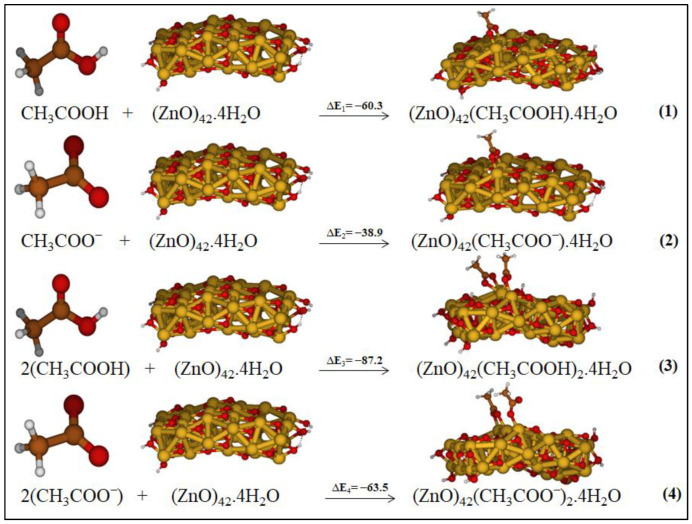
Investigated reactions considering the most stable adsorption systems as products in low-polarity medium. The relative energies are reported in kcal/mol.

**Figure 3:**
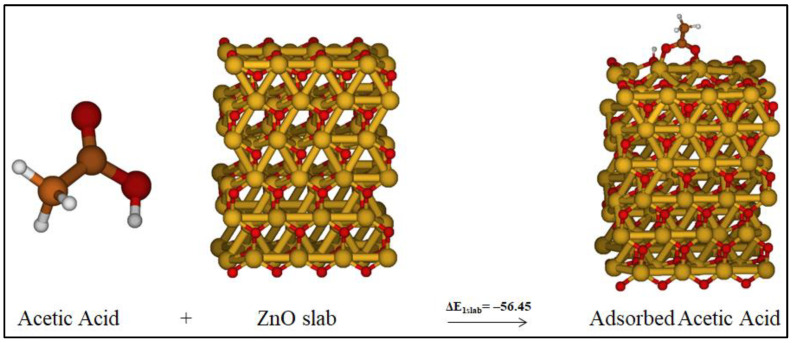
Reaction 1 considering periodic slab calculations.

To further validate our cluster approach, we considered reaction 1, Figure 2, by optimizing the involved species by using a periodic slab approach; see Figure 3. The acetic acid is attached to ZnO surface through a Zn−O bond with a distance range of 1.96–2.028 Å. The obtained results are compatible with those of the cluster approach where ΔE_1slab_ = −56.5 kcal/mol, thus confirming the adequacy of the model in studying this kind of reactivity pathway. 

### 3.2. Zn Carboxylation Reactions

In explaining the degradation phenomena of ZnO pigment in oil paintings, it has been recently observed through FTIR spectroscopy that in aged oil paints, the oil paint binding medium adopts an ionomer-like system when the metal ions bind to the carboxylate groups of the polymerized oil network [6,16,45,46,47]. A proposed degradation mechanism involves the releasing of metal ions from the pigment into the binding medium and subsequent association to carboxylate groups attached to polymer networks [17,18]. Following this hypothesis, zinc ions migrate from ZnO during paint drying and bind to the carboxylate groups [19]. The formation of zinc carboxylate complexes represents an intermediate phase during oil drying, which results in the final appearance of metal soap [13].

**Figure 4:**
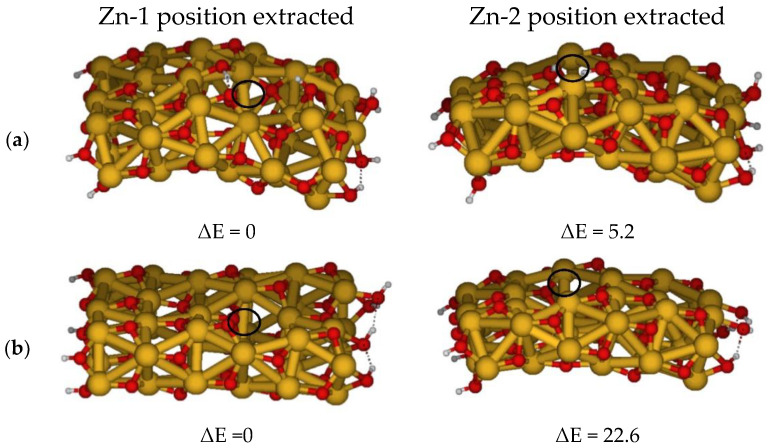
Optimized geometries of ZnO cluster by changing the position of Zn-hole on the surface leaving it protonated (**a**) or deprotonated (**b**), along with the relative energies in kcal/mol.

**Figure 5:**
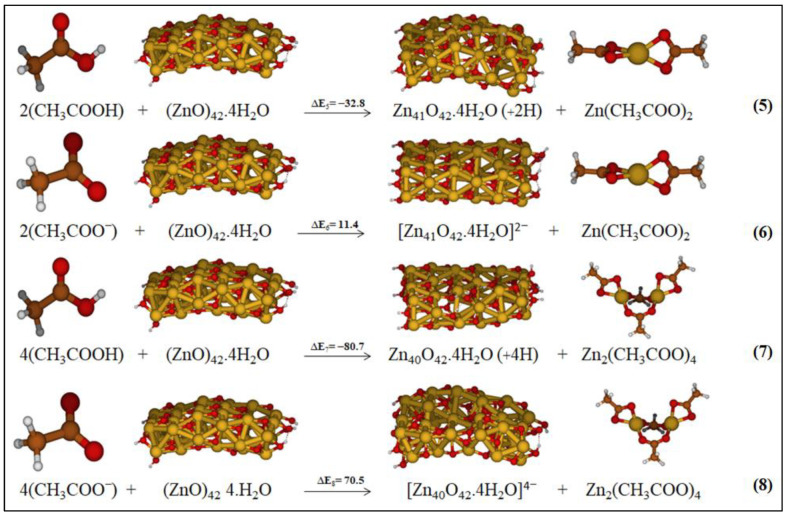
Investigated reactions considering the formation of Zn complexes as products in low-polarity medium. The relative energies are reported in kcal/mol.

We focused on this degradation step, namely the formation of Zn(AcO)_2_ from the released Zn ion from the surface of ZnO and the two acetate units. Starting from the same reactants, the ZnO cluster and acetic acid/acetate, the products should be: (i) the ZnO cluster with a Zn-vacancy on the surface, leaving it protonated, and indicating the transfer of protons from acetic acid or deprotonated in combinations with the acetate or acetic acid depending on the formed Zn complex (see Figure 4), and (ii) the Zn-dicarboxylate (see Figure 5, reactions 5 and 6). 

We optimized the two ZnO clusters with a Zn vacancy and two protons on the surface by exploring different positions of the released ion, which we indicated as Zn-1 and Zn-2 (see Figure 4a). The corresponding optimized structures with no added protons on the surface, referring to the reaction with two acetates, are reported in Figure 4b. The ZnO cluster labelled Zn-1 is the most stable by 5.2 kcal/mol and 22.6 kcal/mol, respectively; see Figure 4. 

When we consider the reaction between ZnO and four acetic acid/acetate Zn_2_(AcO)_4_, the reaction product is a ZnO cluster with two Zn-vacancies and with four protons/no protons on the ZnO surface. Following the same procedure used before, different geometries of the ZnO cluster with two Zn-vacancies on the surface have been optimized in vacuo, changing the positions of Zn released atoms and the related protons (see SI, Figure S4). The most stable optimized structure in low-polarity medium is reported in Figure 5 and it will be one of the products of reaction 7 (Figure 5). We repeated the geometry optimization of the ZnO cluster with two Zn vacancies in different positions and no protons on the surface, associated with the reaction with acetate. The most stable optimized structure will be one of the products of reaction 8 (see Figure 5). 

All the investigated reaction pathways are summarized in Figure 5 along with the associated reaction energies, ΔE, computed in low-polarity medium. The reaction energies computed in all the different considered media are reported in SI, Table S2.

Reactions 5 and 7 involving acetic acids are exothermic in all the considered media, being ΔE_5_ = −32.8 kcal/mol and ΔE_7_ = −80.7 kcal/mol. On the other hand, reactive pathways 6 and 8 having the acetates as reactants are endothermic, ΔE_6_ = 11.4 kcal/mol and ΔE_8_ = 70.5 kcal/mol, by referring to a generic solution (ε = 5). These values are probably overestimated due to the neglect of charge balance on the ZnO surface which should be at least partially present in the realistic system. From our calculations, we can conclude that the reactions involving acetic acid are favored with respect to those with acetate. In vacuo, we also considered the formation of a Zn complex in which the two acetic acids coordinate to the metal not transferring the protons to the ZnO surface. This reaction in vacuo was computed as highly endothermic, essentially due to the instability of the Zn complex with respect to Zn-diacetate. Further calculations in solution were been performed (see SI, Figure S3).

**Figure 6:**
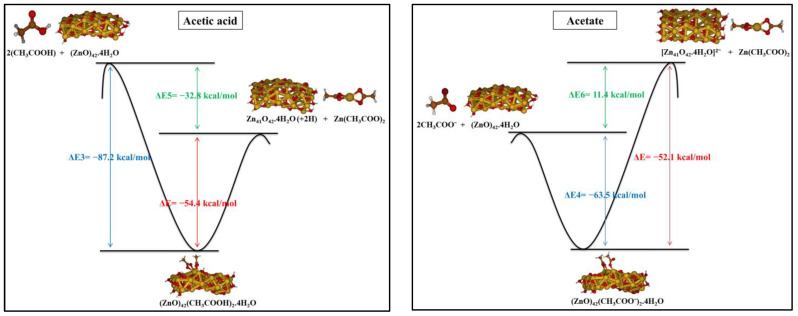
Reaction pathway of acetic acid/acetate and ZnO cluster forming metal soap and initiated by the adsorption of acetic acid/acetate on the surface of ZnO.

By comparing the two studied reaction pathways, we found that the adsorption of acetic acid/acetate on the ZnO surface is thermodynamically favored over the release of Zn ions from the surface to form Zn complexes. These results are consistent with the hypothesis that the reaction pathway leading to the formation of metal soaps is initiated by the adsorption of carboxylic acid/carboxylate on the surface of ZnO cluster (see Figure 6). This adsorption presents a pathway from the global reaction that leads to metal soap formation. 

### 3.3. Acetic vs. Hexanoic Acid Reactivity

**Figure 7:**
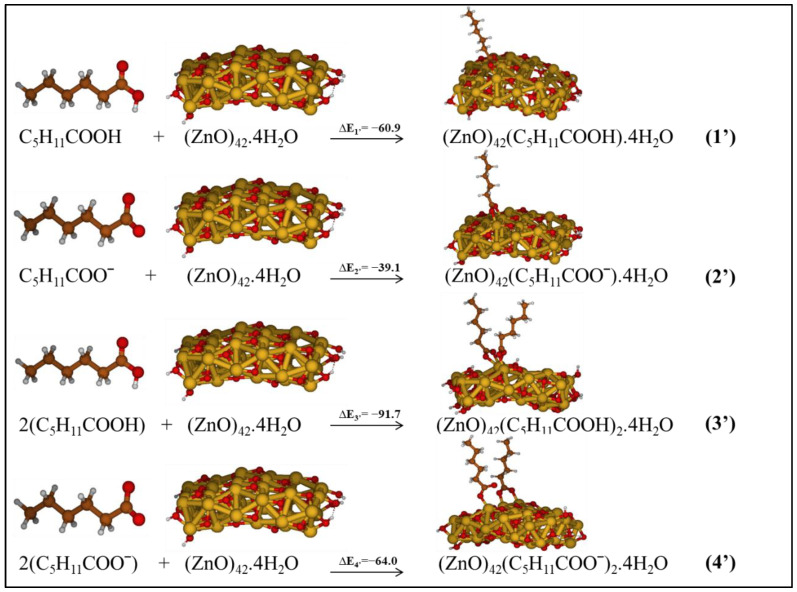
Investigated reactions considering adsorption systems as products in low-polarity medium. The relative energies are reported in kcal/mol.

Following the same strategy applied above, we considered longer chains of carboxylic acids to model the effect of the chain of the oil components in the pigment degradation. As an example, linseed oil, a widely employed medium in oil pigments, contains α-linolenic acid, oleic acid, linoleic acid and small percentages of the saturated palmitic and stearic acids, of chemical formula C_16_H_32_O_2_ and C_18_H_36_O_2_, respectively. We limited our chain’s length to C_6_H_12_O_2_ units, which is a balance between a realistic saturated carboxylic acid and the size of our ZnO cluster. Starting from hexanoic acid/hexanoate and ZnO cluster as reactants, systems in which one or two molecules of hexanoate bind to the zinc of ZnO cluster through a bridged bidentate mode, with or without proton transfer, were considered, as reported in Figure 7. The hexanoic acid adsorbs on two adjacent Zn centers consistently to the acetic acid by a bridged bidentate configuration and in a dissociative adsorption mode. The optimized geometry of one or two molecules of hexanoic acid chemisorbed to ZnO presents Zn–O bond distances ranging between 2.0 and 2.02 Å, analogously to the adsorption of acetic acid/acetate on the ZnO cluster. 

We repeated the geometry optimization for the hexanoate species, considering one hexanoate and two molecules of hexanoate on ZnO. For the latter system, the geometry changed by going from vacuo to solution phases. In a similar way to the previous results with acetic acid, in the solvent geometries one Zn–O bond was detached from the surface.

We focused on the reactions which lead to the most stable hexanoic acid@ZnO and hexanoate@ZnO in a low-polarity medium involving one molecule of hexanoic acid/hexanoate (reactions 1’ and 2’) and two molecules of hexanoic acid/hexanoate (reactions 3’ and 4’); all these reactions are schematized in Figure 7 together with the related ΔE.

Reactions 1’–4’ are exothermic; we found that the adsorption systems associated with hexanoic acid are more favored than those adsorbing hexanoate, which confirms the results obtained for acetic acid. Furthermore, the energetics associated with hexanoic acid/hexanoate show a similar trend as found for acetic acid/acetate. However, the reaction energies ΔE_5’_ and ΔE_7__’_ were found to be larger than ΔE_5_ and ΔE_7_, by ca. 5 and 11 kcal/mol, respectively, the analogous ones involving the acetic acid. An appreciable increase in energy, 4.5 kcal/mol, going from the acetic acid to the hexanoic one was also computed for the coordination of two acids on the same Zn center, reactions 1’ and 3’. The increase in the exothermicity of these reactive steps with hexanoic acids is related to the increase in the electron-donating capability of the longer chain. Therefore, we might conclude that the degradation is favored by longer chains of carboxylic acids, such as those composing linseed oil, which is one of the widely used binders in oil paintings. Acetic acid remains, however, a good model to individuate the main reactive steps of the formation of Zn complexes. 

**Figure 8:**
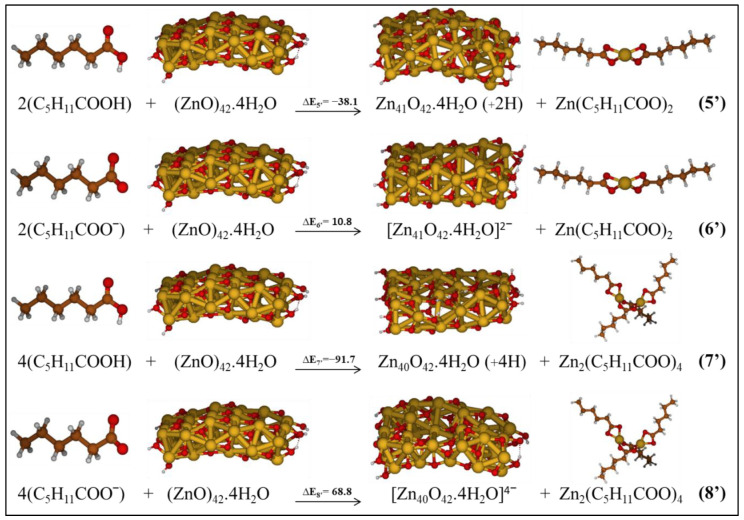
Investigated reactions considering the formation of Zn complexes as products in low-polarity medium.

According to the most stable optimized systems studied in the previous part, we investigated the reactions related to the formation of hexanoate Zn complexes, which are schematized in Figure 8. The interaction of the ZnO cluster and hexanoic acid/hexanoate, respectively, reactions 5’ and 6’, produces: (i) the ZnO cluster with a Zn-hole on the surface, leaving it protonated and indicating the transfer of protons from hexanoic acid, or deprotonated in combination with the hexanoate, and (ii) the Zn-dihexanoate. When we consider the complex of two zinc ions attached to four hexanoate as the final product of the reaction between ZnO and hexanoic acid/hexanoate, the other product of the reaction is a ZnO cluster with two Zn-holes and with four protons/no protons on the ZnO surface, as represented by reactions 7’ and 8’ in Figure 8. 

The energy values are reported in Figure 8 and refer to the solvent with ε = 5. Reactions 5’ and 7’, involving two or four hexanoic acids, are exothermic in all the considered media, ΔE_5’_ = −38.1 kcal/mol and ΔE_7’_ = −91.7 kcal/mol. On the other hand, reactive pathways 6’ and 8’ having the hexanoate as reactant are endothermic, ΔE_7’_ = 10.8 kcal/mol and ΔE_8’_ = 68.8 kcal/mol by referring toa low polarity medium. It is interesting to note that Zn complexes involving two Zn centers are favored with respect to Zn-dicarboxylates, reaction **(7’)** vs **(5’)**, consistently to the formation of Zn soaps in paintings where ZnO pigment is present. A bond analysis using QTAIM tools [48,49,50] would probably suggest the reason for the different stabilities. Here, we can only suppose that the different donor strengths of short/long alkyl chains, together with steric interactions, may be at the origin of the calculated differences. 

**Figure 9:**
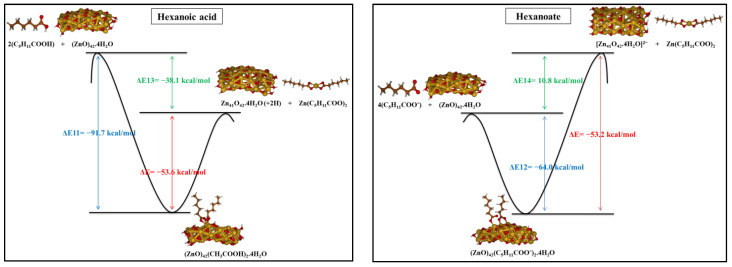
Reaction pathway of hexanoic acid/hexanoate and ZnO cluster forming metal soap and initiated by the adsorption of hexanoic acid/hexanoate on the surface of ZnO.

We observed that the adsorption of hexanoic acid/hexanoate on the ZnO surface is favored over the release of Zn ion from the surface to form Zn complexes (see Figure 9), in agreement with the results obtained for acetic acid. These two investigated reactions represent a simplified model of the key steps of the more complex ZnO degradation pathways.

## 4. Conclusions

Starting from small models of ZnO clusters and carboxylic acid/carboxylate, interesting results were obtained after the optimization of different geometries using the DFT method. Reactions between the ZnO cluster and the acetic acid/acetate as well as the adsorption of the acid/acetate on the cluster surface were investigated. In addition, more realistic models with more extended ZnO clusters and longer chains of carboxylic acids/carboxylate were considered.

Thermodynamically, it is effectively favorable to extract the zinc atom from the ZnO cluster to form metal soap. This reaction pathway is initiated by the adsorption of the carboxylic acid/carboxylate on the surface of the pigment, which is more favored.

Longer alkyl chains of carboxylic acids do not have a significant effect on the cluster when comparing the results to those of acetic acids from structural and energetic points of view. Thus, even a small model of carboxylic acid is quite good at reproducing the properties of the studied systems in which reasonable results were obtained.

The cluster approach was double checked using a slab approach. Thus, we can explore different models using slab and periodic calculations. Moreover, we can study defects that could catalyze these degradation reactions.

## Data Availability

Not applicable.

## Supplementary Materials

The following supporting information can be downloaded at: https://www.mdpi.com/article/10.3390/molecules27113362/s1; Figure S1: Optimized geometry of bridged bidentate adsorption configuration involving two different position of Zn centers in the adsorption. Figure S2: Optimized geometries of possible adsorption systems in low polarity medium, considering one acetate (a) or two acetates (b) on the surface, and the relative energy difference between the configurations in kcal/mol. Figure S3: Investigated reactions in vauo considering the formation of a Zn complex with the two acetic acids coordinate to the metal and the ZnO surface deprotonated. The relative energy is reported in kcal/mol. Figure S4: (a) Optimized geometries of different positions for two released Zn ions from the surface of ZnO cluster leaving it protonated and the relative energy between these geometries in kcal/mol; (b) Optimized geometries of different positions for two released Zn ions from the surface of ZnO cluster leaving it deprotonated, and the relative energy between these geometries in kcal/mol. Table S1. Formation energies in kcal/mol for the investigated reactions reported in Figure 3 in vacuum and solution phases. Table S2. Formation energies in kcal/mol for the investigated reactions corresponding to Figure 5 in vacuum and solution phases. Table S3. Formation energies in kcal/mol for the investigated reactions corresponding to Figure 6 in vacuum and solution phases. Table S4. Formation energies in kcal/mol for the investigated reactions corresponding to figure 7 in vacuum and solution phases.

## Sample Availability

Samples of the compounds are not available from the authors.
