# Supplementary material for: Modelling the Interaction between Carboxylic Acids and Zinc Oxide: Insight into Degradation of ZnO Pigments
Source: Molecules. 2022 May 24;27(11):3362. doi: 10.3390/molecules27113362 (PMC9181975; doi:10.3390/molecules27113362)
Supplement: Supplementary file 1 [file molecules-27-03362-s001.zip › molecules-1715052-supplementary.pdf]

Modelling the Interaction between Carboxylic Acids and Zinc Oxide: Insight into Degradation of ZnO Pigments

Jihan Lubani,[a,b] Filippo De Angelis, [a,b,c,*] Daniele Meggiolaro,[b] Laura Cartechini,[b] Simona Fantacci,[b,*]

[a]*Department of Chemistry, Biology and Biotechnology, University of Perugia, Via Elce di Sotto 8, 06123, Perugia, Italy.*

[b]*Computational Laboratory for Hybrid/Organic Photovoltaics (CLHYO), Istituto CNR di Scienze e Tecnologie Chimiche "Giulio Natta" (CNR- SCITEC), Via Elce di Sotto 8, 06123, Perugia, Italy.*

[c] *Department of Natural Sciences & Mathematics, College of Sciences & Human Studies, Prince Mohammad Bin Fahd University, Saudi Arabia.*

Supporting information

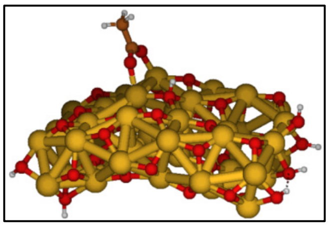

Figure S1. Optimized geometry of bridged bidentate adsorption configuration involving two different position of Zn centers in the adsorption.

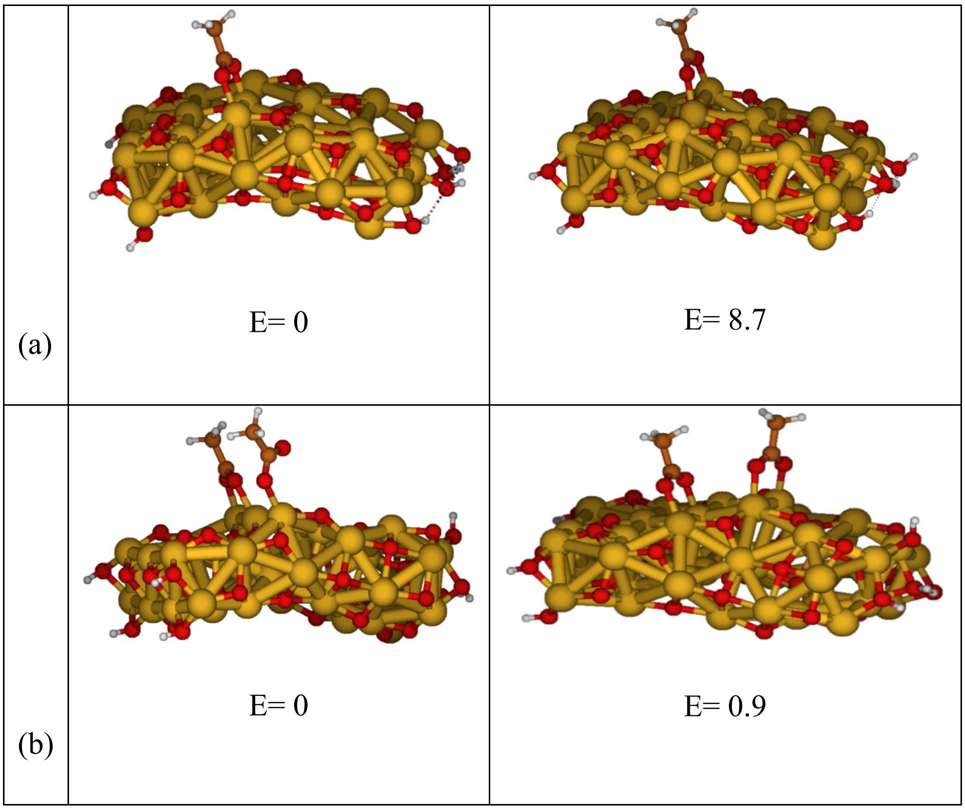

Figure S2. Optimized geometries of possible adsorption systems in low polarity medium, considering one acetate (a) or two acetates (b) on the surface, and the relative energy difference between the configurations in kcal/mol.

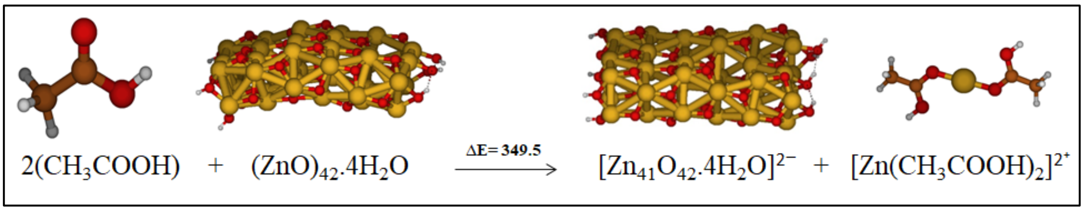

Figure S3. Investigated reactions in vauo considering the formation of a Zn complex with the two acetic acids coordinate to the metal and the ZnO surface deprotonated. The relative energy is reported in kcal/mol.

Zn(1-3)- extracted position	Zn(1-2)-extracted position	Zn(2-3)- extracted position
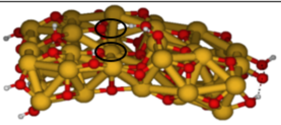 **$E_{(vacuo)}$= 41.7**	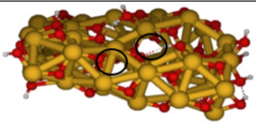 **$E_{(vacuo)}$=3.8**	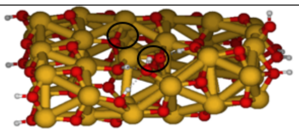 **$E_{(vacuo)}$= 0**
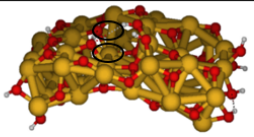 **$E_{(vacuo)}$=7.5**	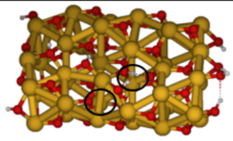 **$E_{(vacuo)}$=34.1**	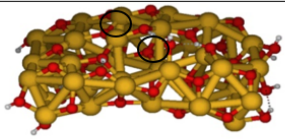 **$E_{(vacuo)}$=10.3**

(**a**)

Zn(1-3)- extracted position	Zn(1-2)-extracted position	Zn(2-3)- extracted position
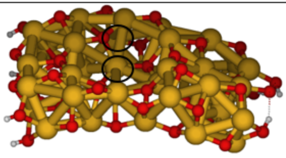 **$E_{(vacuo)}$= 0**	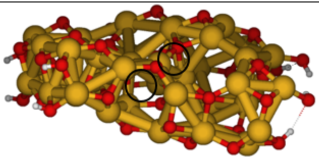 **$E_{(vacuo)}$=62**	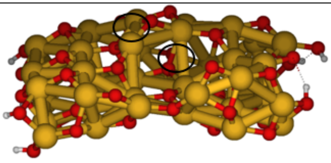 **$E_{(vacuo)}$= 23**

(**b**)

Figure S4. (**a**). Optimized geometries of different positions for two released Zn ions from the surface of ZnO cluster leaving it protonated and the relative energy between these geometries in kcal/mol. (**b**) Optimized geometries of different positions for two released Zn ions from the surface of ZnO cluster leaving it deprotonated, and the relative energy between these geometries in kcal/mol.

ΔE (kcal/mol)	VACUUM	ε = 5	WATER
**Reaction 1**	−96.0	−60.3	−46.5
**Reaction 2**	−104.7	−38.9	−25.4
**Reaction 3**	−123.4	−87.2	−76.6
**Reaction 4**	−142.6	−63.5	−43.6

Table S1. Formation energies in kcal/mol for the investigated reactions reported in Figure 3 in vacuum and solution phases.

ΔE (kcal/mol)	VACUUM	ε = 5	WATER
**Reaction 5**	−40.2	−32.8	−29.1
**Reaction 6**	−38.3	11.4	27.8
**Reaction 7**	−95.1	−80.7	−75.1
**Reaction 8**	84.1	70.5	72.7

Table S2. Formation energies in kcal/mol for the investigated reactions corresponding to figure 5 in vacuum and solution phases.

ΔE (kcal/mol)	VACUUM	ε = 5	WATER
**Reaction 1'**	−97.1	−60.9	−48.6
**Reaction 2'**	−94.1	−39.1	−24.6
**Reaction 3'**	−134.0	−91.7	−69.0
**Reaction 4'**	−140.1	−64.0	−45.1

Table S3. Formation energies in kcal/mol for the investigated reactions corresponding to Figure 6 in vacuum and solution phases.

ΔE (kcal/mol)	VACUUM	ε = 5	WATER
**Reaction 5'**	−51.3	−38.1	−32.5
**Reaction 6'**	−35.5	10.8	27.0
**Reaction 7'**	−116.6	−91.7	−77.6
**Reaction 8'**	90.5	68.8	75.5

Table S4. Formation energies in kcal/mol for the investigated reactions corresponding to figure 7 in vacuum and solution phases.
